# Telemedicine During COVID-19 Pandemic: Lesson Learned from the Lazio Region Infectious Diseases and Emergency Department Network

**DOI:** 10.1007/s10916-022-01887-z

**Published:** 2022-12-07

**Authors:** Gaetano Maffongelli, Nazario Bevilacqua, Serena Vita, Tommaso Ascoli Bartoli, Angela Corpolongo, Domenico Benvenuto, Tiziana Chiriaco, Giuseppe Spiga, Sergio Ribaldi, Valentina Zirretta, Giuseppe Ippolito, Francesco Nicola Lauria, Francesco Vaia, Emanuele Nicastri, The COVID Group of the Lazio Region, Andreoni Massimo, Angelini Daniele, Bertazzoni Giuliano, Betti Antonio, Bonfini Rita, Casinelli Katia, Caterini Luciano, Cedrone Claudia, Cipollone Lorena, Cristofari Fabrizio, Curti Simona, Dal Piaz Rita, Daniele Paolo, Donati Ugo, Fantoni Massimo, Ferri Enrico, Franceschi Francesco, Gugliemelli Emanuele, Iorio Monica, Lapiccirella Paola, Lazzaro Marco, Lichtner Miriam, Magnanti Massimo, Mancini Flavio, Manetti Luca Luigi, Marchili Mauro, Masella Alessandro, Mastroianni Claudio Maria, Mellacina Mario, Miconi Roberto, Mirante Enrico, Nucera Paolo, Paganelli Carla, Pagnanelli Adolfo, Petrolino Maria, Piccolo Carlo Gaetano, Pomes Michele, Pugliese Francesco Rocco, Ricciuto Giulio Maria, Romanelli Antonio Filippo, Ruggieri Maria Pia, Saggese Maria Paola, Sambuco Federica, Sighieri Cinzia, Soleo Flavio, Susi Beniamino, Timpone Sergio, Travaglino Francesco, Urbano Ettore, The Bed Manager Group at INMI Spallanzani, Andrea Antinori, Amina Abdeddaim, Tommaso Ascoli Bartoli, Francesco Baldini, Rita Bellagamba, Nazario Bevilacqua, Evangelo Boumis, Marta Camici, Alessandro Capone, Emanuela Caraffa, Adriana Cataldo, Stefano Cerilli, Carlotta Cerva, Pierangelo Chinello, Stefania Cicalini, Angela Corpolongo, Alessandra D′Abramo, Maria Grazia De Palo, Federico De Zottis, Virginia Di Bari, Francesco Di Gennaro, Gianpiero D′Offizi, Davide Donno, Francesca Faraglia, Vincenzo Galati, Roberta Gagliardini, Saba Gebremeskel Tekle, Maria Letizia Giancola, Guido Granata, Elisabetta Grilli, Fabio Iacomi, Luciana Lepore, Raffaella Libertone, Laura Loiacono, Andrea Mariano, Ilaria Mastrorosa, Valentina Mazzotta, Paola Mencarini, Annalisa Mondi, Silvia Mosti, Maria Musso, Pasquale Noto, Sandrine Ottou, Claudia Palazzolo, Fabrizio Palmieri, Carlo Pareo, Nicola Petrosillo, Carmela Pinnetti, Paolo Migliorisi Ramazzini, Alessia Rianda, Silvia Rosati, Laura Scorzolini, Fabrizio Taglietti, Chiara Taibi, Roberto Tonnarini, Simone Topino, Alessandra Vergori, Laura Vincenzi, Ubaldo Visco-Comandini, Pietro Vittozzi, Mauro Zaccarelli

**Affiliations:** 1 National Institute for Infectious Diseases “Lazzaro Spallanzani” IRCCS via Portuense 292, 00149 Rome, Italy; 2 Local Health Autority Roma 3, Rome, Italy; 3 Giannina Gaslini Institute, 16147 Genova, Italy; 4 Lazio Health Regional Health System, Rome, Italy; 5 Laziocrea associate company of the Lazio Region, Rome, Italy

**Keywords:** COVID-19, Telemedicine, Infectious Diseases, Surveillance

## Abstract

Telemedicine and teleconsultation can be powerful and useful tools for patients to hamper the physical barriers to access to health care services during COVID-19 pandemic. We describe the teleconsultation (TC) model in the Lazio Region. It uses a hub-and-spoke network system on geographic regional basis using a web based digital platform, termed ADVICE with the aim to connect regional Emergency Departments (EDs) and Infectious Diseases (ID) acute and critical care settings for patients with acute ID syndrome. Between January 2020 and June 2021, the ADVICE platform received 18.686 TCs: of them, 10838 requests (58%) were for ID TCs in 7996 patients, followed by 2555(13%) requests for trauma, 2286(12%) for acute complex syndrome and 1681 (8%) for Stroke TCs. Three quarter of ID TCs were requested for SARS-COV-2 infection, followed by sepsis management in 7% and tuberculosis in 6%. In 5416 TCs, 68%, diagnostic investigations and therapeutic prescriptions were recommended before admission, in 1941 TCs, 24%, the recommendation was patient admission and in 608 TCs, 7%, was to discharge patient at home. Telemedicine have ensured high-profile consultations for ID patients and during COVID-19 the use of this resource optimized clinical patient management.

## Introduction

During the COVID-19 outbreak, telehealth technology has been recognized to play a key role in the global response to the spread of SARS-CoV-2 infection. Indeed, it allowed physicians to deliver, via information technologies (IT), health services to patients with limited access to health care services during the quarantine [1].

Telemedicine is the use of telecommunications technology for medical diagnosis and patient care [2]. It is possible to organize specialized advice from a distance by planning an electronic consultation (e-consult). In the emerging field of telemedicine, teleconsultation (TC) is defined as synchronous or asynchronous consultation using information and communication technology to omit geographical and functional distance. Its goals are for diagnostics or treatment between two or more geographically separated health providers [2]. Referring providers send a consultation request to specialists, who may respond by answering the consult question, requesting additional information, and/or indicating diagnostic and therapeutic prescriptions [3]. For a novel infection such as COVID-19, where natural history and optimal clinical management are rapidly evolving, information must be rapidly synthesized, and Infectious Disease (ID) consultations can provide support by performing case-by-case evaluations [4].

**Fig. 1:**
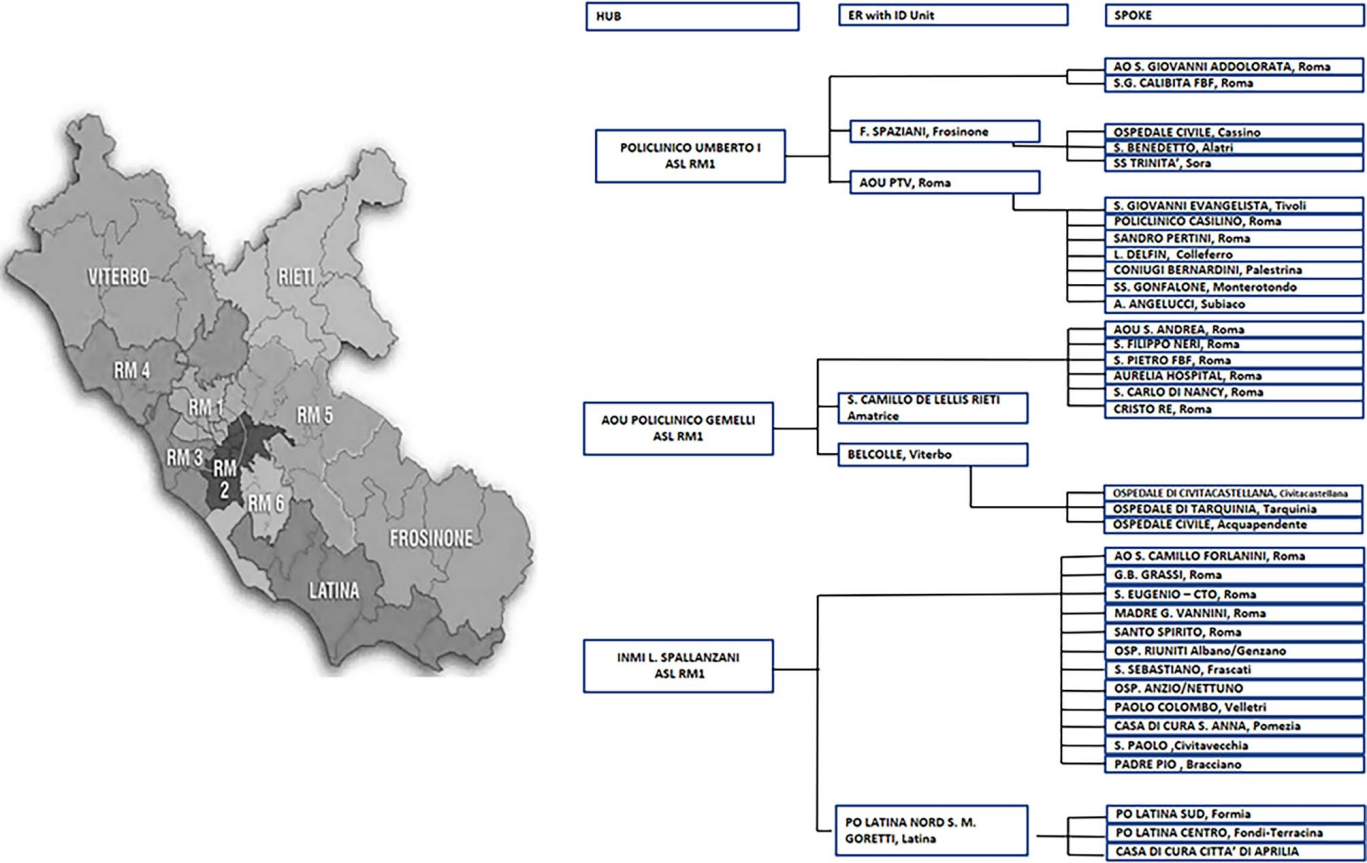
Model of the Infectious Disease emergencies Network in Lazio Region

The Lazio region is one of the 20 administrative regions of Italy and is in the central peninsular area of the country and comprises a land area of 17,242 km^2^. It has 5,864,321 inhabitants, most of them (4,353,738) resident in the metropolitan city of Rome. Rome incorporates the small enclave of Vatican City [5]. In 2010, a Lazio regional law established the legal framework of the regional network of ID units, but only in December 2015, at the time of the last catholic Jubilee, the regional health authorities officially instituted a network of all the 47 regional Emerging Departments (EDs) with the eight ID units operating by a hub-and-spoke network system on a geographic basis (Fig. 1). Since January 2019, regional health authorities have provided all regional EDs with a web-based telemedicine platform system termed ADVICE to obtain remote teleconsultations [6–9]. Through the ADVICE system is possible to ask for different specialist TCs: trauma and polytrauma, complex acute syndromes for poly-pathological patients (CASPP) demanding for multidisciplinary medical services, neurological clinical disorders requiring an urgent assessment by a stroke unit, cardiological emergencies requiring cardio surgical evaluation and pediatric emergencies.

The aim of this report is to describe the experience of telemedicine using the ADVICE digital platform for ID TC service in all EDs of the Lazio region during the COVID-19 outbreak between January 2020 and June 2021.

## Material and methods

We analyzed ID TC requests sent by EDs of peripheral hospitals through ADVICE web-platform, during the period between January 2020 and June 2021. We collected data regarding ED of origin, gender, age of patient, reason for physician request, outcome of counseling, and time between request and release of counseling.

Briefly there are three major ID units operating on call, 24 h per day and 7 days per week, play the role of primary hubs: the Sapienza University Hospital, the Agostino Gemelli Catholic University Hospital and the Lazzaro Spallanzani National Institute for Infectious Disease (INMI), all based in Rome, Italy (Fig. 1). The INMI Hub plays the role of the clinical coordination of the network and the INMI ID physician on call is the bed manager of the regional ID network.

The general data protection officer of the Lazio region and of the Italian data protection authority approved the use of the ADVICE platform for sharing clinical data among health care workers.

Briefly, the ED physician requesting the consultation at spoke peripheral level, produces a brief electronic report including medical history, vital signs and respiratory data, blood tests and radiological images of the patient throughout the ADVICE platform, and eventually, providing additional details by phone, upon request. The ID specialist at hub level is committed to provide an evidence-based, standardized diagnostic and therapeutic approach to patients with acute infectious syndrome, suggesting appropriate infection control measures, referring patients to the acute or critical care ID unit, or, alternatively, discharging them home or in an outpatient setting. The ID specialist formally notifies the ED physician of the closure of the consultation. Eventually, INMI is one of the leading Italian COVID-19 reference centers and, since March 2020, weekly web platform COVID-19 seminars on epidemiologic, clinical, and diagnostic topics have been scheduled for all regional ED physician, intensive care and acute ID consultants.

Finally, this is a cross-sectional survey with no prospective longitudinal data.

## Results

In the interval period between January 2020 and June 2021, the ADVICE platform collected 18.686 requests for TCs from all 47 regional EDs. The 58% (10.834) were ID TCs while the remaining 6726 (42%) were related to patients with non-ID acute syndromes, among them 38% (2555) were trauma TCs followed by 2286 (34%) CASPP TCs, 1681 (25%) Stroke Unit TCs, followed by few remaining cardio surgical and pediatrics TCs.

Of the 47 regional EDs, 37 (78%) actively used the ADVICE platform to ask for an ID consultation, for a total of 10.838 requests (58%) related to 7966 patients.

Regarding the ID TCs, the 75% (8125) of them, were referred to 10 major regional hospitals: 7 of them, located in Rome and 3 in the Latium provinces. Globally, the provinces accounted for the 26% (2.176) of ID TC requests. Table 1 showed the features of ID TCs.

**Table 1:** Infectious Disease teleconsultations features

Total Infectious Disease Teleconsultations (number, n)	10838
Patients evaluated by TCID, n	7966
Mean TC ID per patients, day	1,4
Median TC ID per month, n	497 (range 17:2124)
Time to process ID TC, minutes, mean (min–max)	73 (10–240)
N° TCs for CoVID 19, pts (%)	6054 (76%)
N° TCs for sepsis, pts (%)	797 (7%)
N° TCs for AIDS/HIV, pts (%)	398 (4%)
N° TCs for tubercolosis, pts (%)	478 (6%)
N° TCs for Tropical Diseases or CNS Infections,pts (%)	239 (3%)
Final raccomandations TC ID Diagnostic investigations and therapeutic prescriptions, pts (%)	5416 (68%)
Admission to hospital, pts (%)	1941 (24%)
Discharge to home, pts (%)	608 (7%)

*TC ID* Infectious Disease Teleconsultations, *CNS* central nervous systems, *TC* Teleconsultations, *AIDS* Acquired immune deficiency syndrome, *HIV* Human immunodeficiency virus

**Fig. 2:**
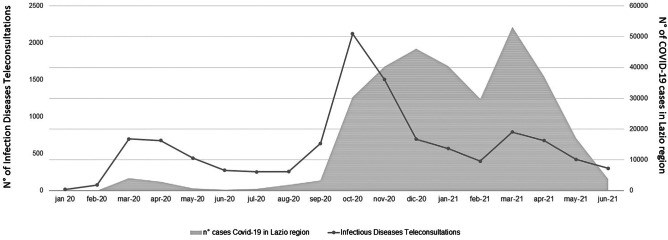
Infectious Disease Teleconsultations and number of COVID-19 in Lazio region trends during the observation period

Patients who underwent ID TCs were mostly male 62% (4938), with a median age of 64 y.o. (min–max, 18–103). The ADVICE platform use progressively increased coinciding with COVID-19 epidemic peaks. This increase was directly related to the increasing number of COVID-19 cases, and particularly, to the different wave peaks of COVID-19 cases in the Lazio region (Fig. 2). A median 497 ID TC (range 17: 2124) per month was reported all over the 18-month period.

ID TCs were mostly requested for patients with acute respiratory syndromes by COVID-19 (6054 clinical cases, 76%). The remaining TCs (797 cases, 7%) were requested for the management of sepsis, followed by tuberculosis (478 cases, 6%), HIV infection/AIDS 398, 4%), tropical infections or central nervous system acute syndromes (239, 3%) as reported in Table 1.

In 5416 out of 7966 cases (68%), further diagnostic investigations and therapeutic prescriptions were recommended by TCs immediately before admission, whereas in 1941 ID TCs (24%), the clinical recommendation was to admit the patient to the ID unit of the hub-and-spoke network system or, in 608 ID TCs, 7%, to discharge the patient to home. The average time for taking charge of the request and for releasing the ID TC was 73 min.

## Discussion

The hub-and-spoke model afford a unique opportunity to maximize efficiencies and effectiveness: from this perspective, telemedicine is a key tool in supporting this model [10–12]. In our region few medical hub responsible for advanced medical services are located in the center of the city while primary care medical services are peripherally spread across an extensive network of secondary spoke sites.

Immediately before the beginning of the 2020 COVID-19 pandemic, the Lazio regional network implemented an ID TC service for all regional EDs, particularly in hospitals with no ID professionals. The use of this web platform during the COVID-19 pandemic has presented an opportunity for implementing telemedicine.

The 78% of the Lazio region spokes have asked for ID consultation and the main reason of TC request was the SARS-CoV-2 infection management, followed by the sepsis management.

This could be probably because the clinical management of COVID patients is constantly evolving and being aware of the state-of-the-art treatments can be challenging for ED physicians and that sepsis is a medical emergency and a significant public health problem that affects millions of people worldwide, representing one of the leading causes of death.

The great number of teleconsultations requests reflect the broadmindedness of ER physicians towards innovative technological tools and the clinical support that having an ID specialist consultation could provide to ED physicians, especially in peripheral areas hospitals. This telemedicine approach has allowed the patient to receive the best clinical management in slightly more than 1 h from the ID TC request. This significantly improved the quality and the reliability of clinical care based on scientific evidence. Moreover, ID teleconsultations allowed the patient to be referred in a short time to hospitalization, to further investigations or to home discharge, ensuring a shorter stay in the ED and thus avoiding crowding.

In ED setting, the immediate taking care of new suspected or confirmed COVID-19 cases is crucial. Indeed, both early recognition of severe patients and identification of the best heath care settings depending on clinical severity (general or critical care units) make resource allocation more efficient. Moreover, it allows to discharge home pauci- or asymptomatic patients using standardized protocols and, finally, to contain the viral circulation among susceptible subjects. In the context of sepsis, having a short time to set antibiotic therapy and an appropriate treatment regimen is already demonstrated to improve clinical outcome [13].

Monkowski et al. already demonstrated the effectiveness of telemedicine in the the setting of infectious diseases [14], moreover, during the SARS-CoV-2 pandemic telehealth has been developed further. In the province of Shandong in China, an ID consulting service has been established through a digital platform cutting time and expenses, while decreasing the risk of spread of SARS-CoV-2 infection by avoiding close contacts with affected patients [15]. Moreover, ID teleconsultation has been also associated with low ICU mortality and low ICU length of stay in patients with sepsis [16, 17].

In the development of ID telemedicine, however, few concerns are to be considered. The lack of digital literacy can be frustrating, but few sessions of training could help to identify simple digital solutions. The management of personal data must be in line with data protection laws especially in the European Union [18, 19]. A delay in releasing ID teleconsultations occurred when untenable patient volumes overwhelmed EDs. However, the number of available ID professionals has increased in response to the surging demand for ID TCs, particularly on night and during holidays. The use of videoconference system integrated in the ADVICE platform is not widely spread. Easy-to-use and inexpensive technological solutions could be the solution to move towards from ID teleconsultation to a tele visit with the patient remote view. Finally, this is a cross-sectional survey with no prospective longitudinal data. In the future, ADVICE platform database could be linked to the regional health care system platform, fully respecting data protection European regulation.

Finally, apart from pandemic scenarios, it is mandatory to take advantage of new digital technologies in the medical setting, that overcoming physical barriers and supporting general or ED physicians in peripheral hospital to have bedside evidence-based clinical advices.

Implementing a standardized telemedicine intervention model will overcome the increasing limitation of human and technical resources, will save time and costs and will help to maintain a high standard of care even in peripheral district.

## Data Availability

Data used to support the findings of this study are available from the corresponding author upon request.

## References

[1] Smith AC, Thomas E, Snoswell CL, Haydon H, Mehrotra A, Clemensen J et al (2020) Telehealth for global emergencies: Implications for coronavirus disease 2019 (COVID-19) [Internet]. Vol. 26, Journal of Telemedicine and Telecare. SAGE Publications. p. 309–13. Available from: 10.1177/1357633X20916567 10.1177/1357633X20916567 PMC7140977 32196391

[2] Anthony B Jnr Use of telemedicine and virtual care for remote treatment in response to COVID-19 Pandemic J Med Syst. 2020 Jun 15;44(7):132. 10.1007/s10916-020-01596-5. 10.1007/s10916-020-01596-5 PMC7294764 32542571

[3] Sethuram C, Helmer-Smith M, Karunananthan S, Keely E, Singh J (2022). C Liddy Electronic consultation in correctional facilities worldwide: a scoping review BMJ Open..

[4] Nguyen CT, Olson G, Pho MT, Lew AK, Pitrak D, Satzman J et al (2020) Automatic ID consultation for inpatients with COVID-19: Point, counterpoint, and a single-center experience [Internet]. Vol. 7, Open Forum Infectious Diseases. Oxford University Press (OUP); Available from: 10.1093/ofid/ofaa318 10.1093/ofid/ofaa318 PMC7454912 33117849

[5] http://www.regione.lazio.it/statistica/it/lazio-in-numeri/popolazione-e-famiglie/popolazione. Last Accessed Apr 2022

[6] Decreto del Commissario ad Acta N U 58, del 12 .07.2010 “ La rete assistenziale delle Malattie Infettive” Available from https://www.inmi.it/bedmanager. Last Accessed Apr 2022

[7] Decreto del Commissario Ad Acta N U00452, del 29.09.2015 “Piano Regionale per la Sorveglianza e la Gestione di Emergenze Infettive durante il Giubileo Straordinario 2015 – 2016 “.Available from https://www.inmi.it/bedmanager. Last Accessed Apr 2022

[8] Decreto del Commissario Ad Acta N. U00540, del 12.11.2015 “Percorso assistenziale per la gestione dei casi con patologia infettiva primaria o associata a comorbidità “ Available from https://www.inmi.it/bedmanager. Last Accessed Apr 2022

[9] Delibera di Giunta regionale del 30 ottobre 2018, n. 626, “Programma di miglioramento e riqualificazione (art. l, comma 385 e ss. Legge 11 dicembre 2016 n. 232) Intervento 2.1 Telemedicina nelle Reti Ospedaliere dell'Emergenza. Attivazione della piattaforma "ADVICE" per il teleconsulto fra centri "Hub" e "Spoke" delle Reti dell'Emergenza, Tempo-dipendenti e delle Malattie Infettive” Available from https://www.regione.lazio.it/sanità Last Accessed Apr 2022

[10] Elrod JK, Fortenberry JL (2017). The hub-and-spoke organization design: an Avenue for serving patients well. BMC Health Serv Res.

[11] Devarakonda S (2016). Hub and spoke model: making rural healthcare in India affordable, available and accessible. Rural Remote Health..

[12] Anderson E, Rinne ST, Orlander JD, Cutrona SL, Strymish JL (2021). VG Vimalananda Electronic consultations and economies of scale: a qualitative study of clinician perspectives on scaling up e-consult delivery Am Med Inform Assoc..

[13] Liu VX, Fielding-Singh V, Greene JD, Baker JM, Iwashyna TJ, Bhattacharya J (2017). GJ Escobar The Timing of Early Antibiotics and Hospital Mortality in Sepsis Am J Respir Crit Care Med..

[14] Monkowski D, Rhodes LV III, Templer S, Kromer S, Hartner J, Pianucci K et al (2019) A Retrospective Cohort Study to Assess the Impact of an Inpatient Infectious Disease Telemedicine Consultation Service on Hospital and Patient Outcomes [Internet]. Clinical Infectious Diseases. Oxford University Press (OUP). Available from: 10.1093/cid/ciz293 10.1093/cid/ciz293 31002338

[15] Song X, Liu X, Wang C (2020) The role of telemedicine during the COVID-19 epidemic in China—experience from Shandong province [Internet]. Vol. 24, Critical Care. Springer Science and Business Media LLC. Available from: 10.1186/s13054-020-02884-9 10.1186/s13054-020-02884-9 PMC7187668 32345359

[16] Young LB, Chan PS, Lu X, Nallamothu BK, Sasson C, Cram PM (2011) Impact of Telemedicine Intensive Care Unit Coverage on Patient Outcomes [Internet]. Vol. 171, Archives of Internal Medicine. American Medical Association (AMA). Available from: 10.1001/archinternmed.2011.61 10.1001/archinternmed.2011.61 21444842

[17] Udeh C, Perez-Protto C, Canfield CM, Sreedharan R, Factora F, Hata JS.^.^ Outcomes Associated with ICU Telemedicine and Other Risk Factors in a Multi-Hospital Critical Care System: A Retrospective, Cohort Study for 30-Day In-Hospital Mortality Telemed J E Health. 2022 Mar 16. 10.1089/tmj.2021.0465. Online ahead of print. 10.1089/tmj.2021.0465 35294855

[18] https://www.garanteprovacy.it/regolamentoue/formazione/ last Accessed July 2021

[19] Li P, Luo Y, Yu X, Wen J, Mason E, Li W et al (2020) Patients’ Perceptions of Barriers and Facilitators to the Adoption of E-Hospitals: Cross-Sectional Study in Western China [Internet]. Vol. 22, Journal of Medical Internet Research. JMIR Publications Inc.; p. e17221. Available from: 10.2196/17221 10.2196/17221 PMC7317627 32525483

